# Mitochondrial Dynamics Decrease Prior to Axon Degeneration Induced by Vincristine and are Partially Rescued by Overexpressed cytNmnat1

**DOI:** 10.3389/fncel.2016.00179

**Published:** 2016-07-19

**Authors:** Gregory W. Berbusse, Laken C. Woods, Bhupinder P. S. Vohra, Kari Naylor

**Affiliations:** ^1^Department of Cellular Physiology and Molecular Biophysics, University of Arkansas for Medical SciencesLittle Rock, AR, USA; ^2^Department of Biology, University of Central ArkansasConway, AR, USA; ^3^Department of Neurology, Yale University School of MedicineNew Haven, CT, USA

**Keywords:** neurodegeneration, mitochondria, Nmnat, fission, fusion, vincristine

## Abstract

Axon degeneration is a prominent feature of various neurodegenerative diseases, such as Parkinson’s and Alzheimer’s, and is often characterized by aberrant mitochondrial dynamics. Mitochondrial fission, fusion, and motility have been shown to be particularly important in progressive neurodegeneration. Thus we investigated these imperative dynamics, as well as mitochondrial fragmentation in vincristine induced axon degradation in cultured dorsal root ganglia (DRG) neurons. CytNmnat1 inhibits axon degeneration in various paradigms including vincristine toxicity. The mechanism of its protection is not yet fully understood; therefore, we also investigated the effect of cytNmnat1 on mitochondrial dynamics in vincristine treated neurons. We observed that vincristine treatment decreases the rate of mitochondrial fission, fusion and motility and induces mitochondrial fragmentation. These mitochondrial events precede visible axon degeneration. Overexpression of cytNmnat1 inhibits axon degeneration and preserves the normal mitochondrial dynamics and motility in vincristine treated neurons. We suggest the alterations in mitochondrial structure and dynamics are early events which lead to axon degeneration and cytNmnat1 blocks axon degeneration by halting the vincristine induced changes to mitochondrial structure and dynamics.

## Introduction

In addition to providing the largest sources of cellular ATP, mitochondria are intimately involved in the regulation of intracellular calcium levels and pH, they serve as oxygen sensors, and are the source of super-oxide radicals (Müller et al., 2005). Current views of mitochondrial dynamics have disproven the traditional notion of singular, randomly dispersed organelles and it is now accepted that mitochondria constitute a population of organelles that are actively transported through the cell, fuse, divide, and undergo regulated turnover (Nunnari et al., 1997; Dedov and Roufogalis, 1999; Chen and Chan, 2005, 2009). Because of these dynamic processes, mitochondria are able to respond to cellular demands, influence cytosolic communication and signaling cascades, be moved to critical subcellular compartments, and finally, assess and respond to mitochondrial fitness (Chen and Chan, 2009). Within the past decade, studies have highlighted the impetus of mitochondrial dynamics in maintaining integral cell and animal physiological processes, influencing function, differentiation, and ultimately affecting survival (Chen and Chan, 2005, 2009). More specifically mis-regulated dynamics- mitochondrial fission and fusion- have been shown to increase reactive oxygen species (ROS), decrease ATP production, as well as detrimentally alter apoptosis (Liesa et al., 2009; Martin, 2012) and mitophagy (Rambold et al., 2011; Shen et al., 2014). These deficits are also associated with numerous neurodegenerative disorders including Parkinson’s, Alzheimer’s, Charcot-Marie Tooth, Amyotrophic Lateral Sclerosis, and Huntington’s diseases (Wang et al., 2005; Press and Milbrandt, 2008; Chen and Chan, 2009; Vohra et al., 2010; Wen et al., 2011; Martin, 2012; Alobuia et al., 2013; Korobova et al., 2013).

Typically preceding clinical symptoms, axonal degeneration is a prominent feature of peripheral neuropathies and neurodegenerative disorders. Wallerian degeneration, a self-destructive process at the distal portion of transected axons, has proven to be a useful model for studying the mechanism of axon degeneration (Wang et al., 2005). Having been shown to occur without activating the caspase family of cysteine proteases, Wallerian degeneration is described as being mechanistically different from neuronal apoptosis triggered by nerve growth factor (NGF) deprivation (Wang et al., 2005; Press and Milbrandt, 2008; Vohra et al., 2010; Wen et al., 2011). Insight into the mechanism of Wallerian degeneration came from a spontaneously occurring mutant mouse strain whose axons survived for weeks post-transection due to the overexpression of a fusion protein (Wld^S^) containing full length nicotinamide mononucleotide adenylyl transferase1 (Nmnat1; Wang et al., 2005). Further investigation revealed that overexpression of Nmnat1, an enzyme required for nicotinamide adenine dinucleotide (NAD^+^) biosynthesis, alone can prevent axon degeneration from physical and chemical triggers (Wang et al., 2005). Catalyzing a key step in NAD^+^ synthesis, Nmnat1 plays an evolutionarily conserved role in neuronal maintenance (Sasaki et al., 2009). Although overexpression of Nmnat1 does not increase the level of NAD^+^ in the neurons, it has been shown to reduce the loss of NAD^+^ in transected axons (Wang et al., 2005; Sasaki et al., 2009). NAD^+^ depletion in transected neurons is decreased in parallel with ATP levels supporting the notion that Nmnat1 acts through local bioenergetics pathways in transected axons (Wang et al., 2005; Press and Milbrandt, 2008; Vohra et al., 2010; Wen et al., 2011).

Mitochondria are implicated in neurodegeneration (Martin, 2012), for example mitochondrial depolarization induces axon degeneration that is independent of classical cell death pathways (Gerdts et al., 2013). Interestingly this form of degeneration can be prevented by depletion of Sarm1 (SARM, sterile α-motif-containing and armadillo-motif containing protein) (Araki et al., 2004; Gerdts et al., 2013; Summers et al., 2014). Additionally, Nmnat1 overexpression prevents axon degeneration induced by mitochondrial toxins (Press and Milbrandt, 2008). Recent work has demonstrated that loss of mitochondrial dynamics prevent mitophagy which is linked to neurodegeneration in mice (Twig et al., 2008; Chen et al., 2015). Finally it has been indicated that activation of the mitochondrial transition pore (mPTP) is a key regulator of axonal degeneration (Barrientos et al., 2011).

At this time the role of mitochondria in neurodegeneration is not completely understood especially the role mitochondrial dynamics may play. Thus we investigated the relationship of mitochondrial dynamics in vincristine induced axon degeneration and Nmnat1 mediated axonal protection. We analyzed mitochondrial dynamics in cultured dorsal root ganglia (DRG) neurons overexpressing cytNmnat1 and treated with vincristine. Vincristine, an anti-cancer drug, is a known stabilizer of microtubules and has been shown to cause peripheral neurotoxicity, likely through alteration of calcium and ATP levels (Canta et al., 2015). The effect of cytNmnat1 overexpression on the rates of mitochondrial fission, fusion, motility, and mitochondrial fragmentation was assessed utilizing confocal laser microscopy at time points associated with axon degeneration—as elucidated from previous studies (Press and Milbrandt, 2008; Vohra et al., 2010; Wen et al., 2011). Our results indicate that during the process of neuronal degeneration, induced by vincristine, fission, fusion and motility rates were decreased and the mitochondria fragmented. More importantly, we show that the neuroprotective protein, cytNmnat1 significantly prevented the alteration of mitochondrial fission, fusion and fragmentation. Most interesting is that our work suggests these alterations occur prior to significant deterioration of the axon.

## Materials and Methods

### DRG Culture

This study was carried out in accordance with the recommendations of PHS Policy on Humane Care and Use of Laboratory Animals, the Guide for the Care and Use of Laboratory Animals, and the policies and procedures of the University of Central Arkansas. The protocol was approved by the UCA Animal Care and Use committee.

Mouse DRG were collected from embryonic day 12.5 (E12.5) CD1 mice embryos. The DRGs from 5 embryos (~200 total DRGs) of CD1 mice were dissociated and resuspended in 50 μl of Neurobasal media (Invitrogen) containing 2% B27 (Invitrogen) and 50 ng/ml NGF (Harlan Bioproducts) per dissected embryo. Suspended DRG neurons were placed as a drop (2 μl) near one end of each well in Lab TekII 4-well chambered cover glass (Nalge Nunc International), which had been previously coated with poly-D-lysine (0.1 mg/ml) and laminin (2–5 ug/ml). The chambered cover glass was then incubated at 37°C at 5% CO_2_ for 15 min before 500 μl of Neurobasal media containing 2% B27, 50 ng/ml NGF, 1 μm 5-fluro-2′-deoxyuridine (Sigma), and 1μm uridine (Sigma) was added to each well.

Vincristine (0.4 μM) was added to 14 days *in vitro* (DIV) cultures to induce axon degeneration.

### Lentiviral Infection of DRG Neurons

Lentiviruses were generated as previously described (Araki et al., 2004). DRG neurons were infected with lentiviruses expressing Ds-Red Mito to track mitochondria and GFP-cytNmnat1 (10^5^–10^6^ infectious units in 1 DIV neurons) (Baloh et al., 2007; Vohra et al., 2010). Control cells were infected with lentivirus expressing Ds-Red Mito and EGFP-only vector. Virus-containing medium was replaced with fresh media at 2 DIV and gene expression was verified via fluorescent microscopy of the EGFP or Ds Red reporter.

### Quantification of Mitochondrial Fission and Fusion in E12.5 CD-1 Mouse DRGs

A Zeiss laser scanning LSM Pascal confocal microscope with 40 × and 63 × objective lenses was used to visualize 0.50 μm thick optical slices of DRG axons with a single plane imaged every 2.97 s for 180 s or until bleaching occurred. Fluorescent images as well as bright field images were collected.

An organelle splitting into two separate entities and remaining that way for the next couple of frames was classified as a fission event. Fusion events were identified when two organelles approached each other, connected, and remained that way for the next couple of frames (Schimmel et al., 2012). To ensure accurate quantification, two approaching organelles, appearing to undergo fusion, which appeared in subsequent frames to have passed by each other—returning to a similar arrangement as in the preceding frames—were not counted as an event; similar precautions were taken when identifying fission events where an organelle that appeared to split into separate organelles only to return to its original conformation in the subsequent frames was not counted.

Rates of fission and fusion were calculated by taking the average number of events/min for each time point at each condition and presented as a mean ± the standard error. Statistical analysis was conducted utilizing Graph Pad Prism version 6.07 and analyzed via non-parametric Kruskal-Wallis with Dunn’s *post hoc*. Statistics generated are from embryos from at least two independent litters, collected on at least two different occasions, except for 96 h, litters were only collected once (3–5 images were collected for each condition/litter), *p*-values less than 0.05 were considered statistically significant.

### Quantifying Mitochondrial Fragmentation in E12.5 CD-1 Mouse DRGs

Zeiss LSM browser was utilized to take a single frame out of two movies from each time point. The lengths and widths of 30 mitochondria were measured under each condition. Fragmentation was determined by the average length to width assessment of the organelles.

Statistical analysis was conducted utilizing a non-parametric multiple comparisons Wilcoxon analysis by JMP statistical analysis software (SAS Institute, Inc.) to compare the measurements from each condition and time point with *p*-values less than 0.05 being considered statistically significant. Comparison of treatments to control at 96 h was carried out in Graph Pad Prism version 6.07 via 1-way ANOVA, Holm-Sidak’s multiple comparisons test.

### Quantifying Mitochondrial Motility in E12.5 CD-1 Mouse DRGs

Kymographs were generated using ImageJ from the single plane time lapse confocal images collected as described above. A region of interest (ROI) was selected in three different areas in the first image of each series, for a minimum of 15 kymographs (approximately 60 mitochondria) for each condition. The ROIs were approximately the same width and length in each instance. The ROIs were converted, stacked, and used to create kymographs, which depict mitochondrial movement specific to the selected ROI. To quantify motility, mitochondrial movement in the kymograph was traced with a line where the slope of the line represents velocity in pixels/time point. These velocities were converted to μm/s.

Statistical analysis was conducted by utilizing Graph Pad Prism version 6.07 to compare velocity measurements within each treatment and across time points with *p*-values less than 0.05 being considered statistically significant. Rates were analyzed via non-parametric Kruskal-Wallis with Dunn’s *post hoc*.

### Scoring Degeneration of Axons

Single frames from every video acquired, as described above, were analyzed for neurodegeneration. A minimum of 50 axons were quantified for all treatments except cytNmnat1 96 h treated axons which only had 32 distinguishable axons. Each axon was classified based on degeneration into five categories and assigned a value between 1 and 5 (adapted from Fang et al., 2014). Axons classified as 5 were fully intact with smooth membrane along the length of the axon (Figure 4B left column). Category 4 were intact axons that had 21–40% of its membrane beginning to bleb (Figure 4B vincristine 24 h frame). Axons that scored a 3 were 41–60% degenerated; these were still intact, had not begun fragmenting, but had extensive blebbing (Figure 4B vincristine 48 h frame). The fourth category, which was assigned a value of 2, were 61–80% degenerated; these axons had begun fragmenting in addition to extensive blebbing of the membranes (Figure 4B vincristine 48 h). Please note: vincristine 48 h treatment images consistently contained both category 2 and category 3 axons. The final category, valued as a 1, were 100% degenerated axons, in these images it was not possible to identify the original number of axons (Figure 4B bottom frame of middle column). Each image was given a score by multiplying the number of axons that fell into each category (i.e., 5 axons in category 5 so 5 × 5 = 25; 2 axons in category 1 so 2 × 1 = 2) followed by these values being added together (i.e., 25 + 2 = 27). For each treatment the image scores were averaged and a two-way ANOVA with *post hoc* Tukey test in Graph Pad Prism version 6.07 was performed.

**Figure 1:**
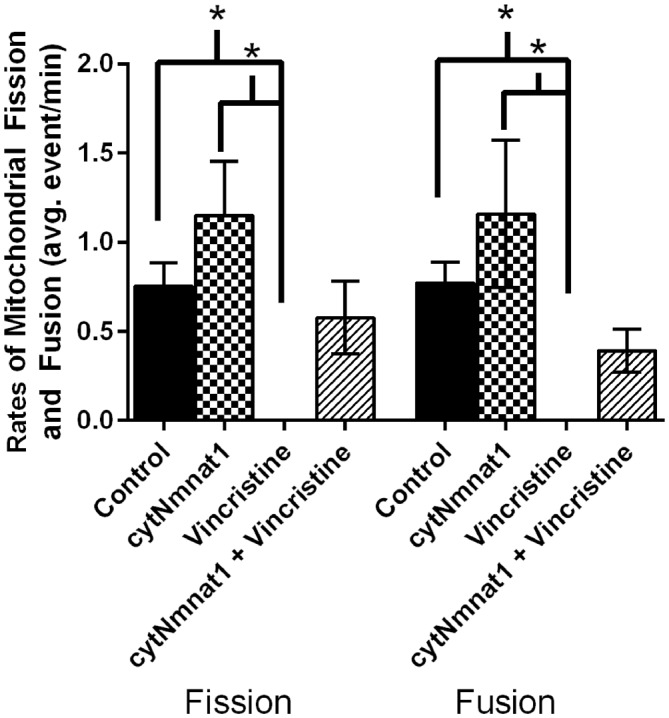
**The average rates of mitochondrial fission and fusion in axons of dorsal root ganglion neuronal cultures at 96 h**. Cultures were either untreated, overexpressed the protective protein GFP-cytNmnat1, treated with 0.4 μM vincristine, or overexpressed GFP-cytNmnat1 and treated with vincristine. The overexpression of cytNmnat1 recovered the fission and fusion processes inhibited by vincristine (*p* < 0.05). * Indicate significance.

**Figure 2:**
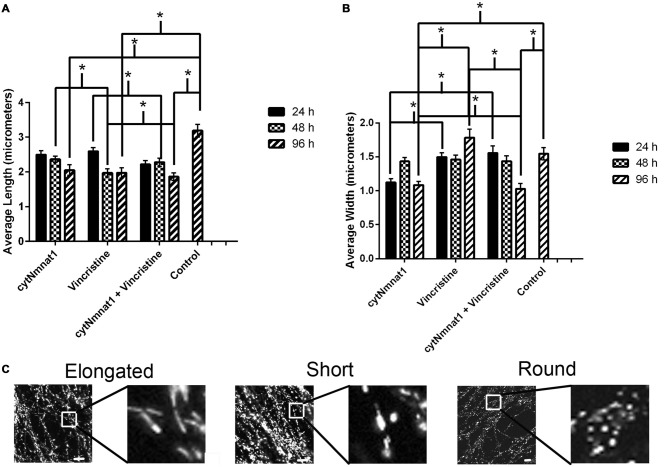
**The length and width of mitochondria for each treatment condition of dorsal root ganglion neuronal cultures over time. (A)** Graph representing the average mitochondrial length for each treatment over time. **(B)** Graph representing the average mitochondrial width for each treatment over time. The mitochondrial length decreased in correlation with an increase in the mitochondrial width in vincristine only treated cells, indicating an increase in fragmentation. The data indicate that overexpression of cytNmnat1 preserves mitochondrial morphology when axon degeneration has been initiated by vincristine (*p* < 0.05). For simplicity only significance between treatments, not over time, is shown. **(C)** Representative mitochondrial images showing elongated, short, and round or fragmented organelles. In general mitochondria with overexpressed cytNmnat1 are elongated but do get shorter over time, while vincristine treated mitochondria become round and fragmented. Scale bar is equivalent to 10 μm. * Indicate significance.

**Figure 3:**
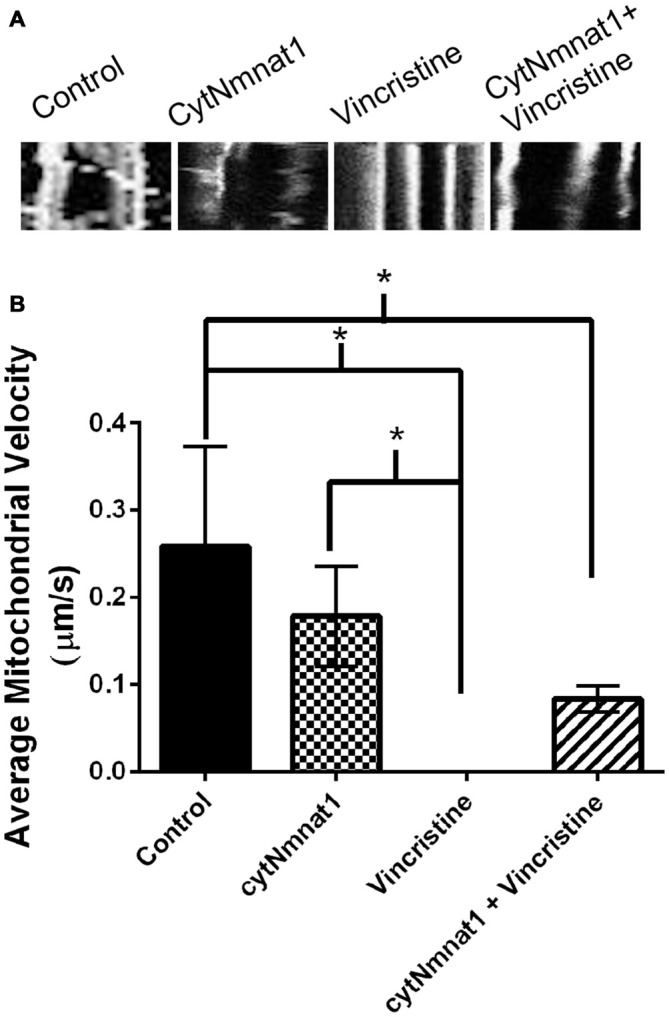
**Mitochondrial velocity for each treatment condition. (A)** Representative kymographs of mitochondria under each treatment. **(B)** Graph representing the average mitochondrial velocity at 96 h post treatment. The overexpression of cytNmnat1 in vincristine treated cells allows the mitochondria to partially regain motility as compared to vincristine treatment alone. * Indicate significance.

**Figure 4:**
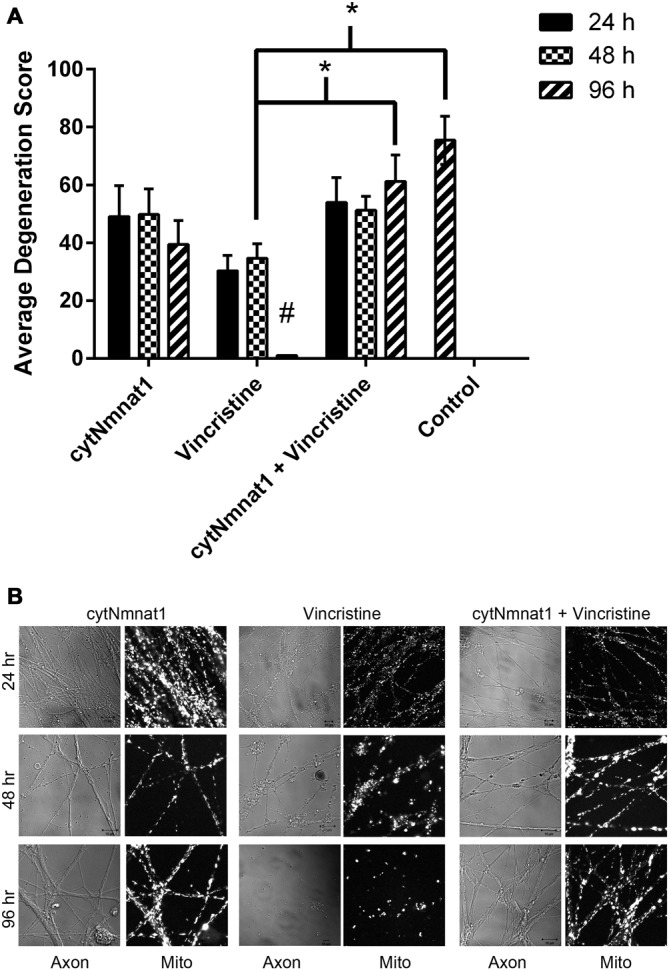
**Degeneration of axons over time**. Axons were assigned a score (see “Materials and Methods” Section) based on their degeneration status. **(A)** Graph of degeneration for each treatment. Vincristine treated axons were degenerated by 96 h, which overexpression of cytNmnat1 prevented (*p* < 0.0001). ^#^ A significant difference when compared to all means, * a significant difference between the paired columns. **(B)** Representative images for each treatment. Fully intact axons are found in all cytNmnat1 treatments. Vincristine treated axons for 24 h typically showed intact axons with blebbing beginning to occur. Extensive blebbing and axon fragmentation can be seen in 48 h vincristine treatments, whereas fully degenerated axons are found in 96 h vincristine treatment. More than 100 axons were analyzed for each treatment except for: 96 h cytNmnat1 overexpression and 96 h cytNmnat1 + vincristine, 30 and 50 axons respectively were quantified.

## Results

### Overexpression of cytNmnat1 in DRGs Maintains Rates of Mitochondrial Fission and Fusion in the Presence of Vincristine, an Inducer of Neurodegeneration

To determine the effect of vincristine induced axon degeneration and cytNmnat1 on mitochondrial fission and fusion, axons were imaged at different time points and the rates of fission and fusion were quantified. Mitochondrial fission rates decreased in the axons of 24 h vincristine treated neurons by 59% compared to cytNmnat1 expressing cultures (Table 1). Similarly, mitochondrial fusion rates also decreased in the axons of vincristine treated neurons by 68% as compared to the cytNmnat1 expressing cells (Table 1). These events were undetectable after 96 h of vincristine treatment (Figure 1, Table 1).

**Table 1:** **Rates of fission and fusion in axons treated with vincristine (events/min)**.

		24 h	48 h	96 h
cytNmnat1	Fission	1.202 ± 0.216	1.049 ± 0.215	0.750 ± 0.226
	Fusion	1.219 ± 0.286	1.136 ± 0.164	1.158 ± 0.413
Vincristine	Fission	0.497 ± 0.117	0.029 ± 0.029	0.000 ± 0.000
	Fusion	0.387 ± 0.111	0.115 ± 0.064	0.000 ± 0.000
cytNmnat1	Fission	0.557 ± 0.135	0.389 ± 0.151	0.509 ± 0.195
+ vincristine	Fusion	0.400 ± 0.136	0.286 ± 0.093	0.390 ± 0.119

To determine if cytNmnat1 will prevent the vincristine induced loss of fission and fusion, rates in vincristine treated axons were compared to rates of fission and fusion in axons overexpressing cytNmnat1 and exposed to vincristine. By 48 h of treatment, fission and fusion rates are recovering; the expression of cytNmnat1 increased these rates by 93% and 60%, respectively, as compared to the axons of vincristine treated neurons (Table 1). This trend shows significant protection by 96 h for both fission and fusion (*p* < 0.05, Figure 1, Table 1). In conclusion, the overexpression of cytNmnat1 recovered the fission and fusion processes in the axons inhibited by vincristine, maintaining the rates at cytNmnat1 expressing culture levels by 96 h.

### cytNmnat1 Overexpression Inhibits Mitochondrial Fragmentation

In order to assess cytNmnat1’s effect on mitochondrial fragmentation the length and width of 30 mitochondria from each time point were measured in the axons of DRG neuronal cultures that were overexpressing cytNmnat1, incubated with vincristine alone, or overexpressing cytNmnat1 + vincristine.

As expected, vincristine treatment fragmented the mitochondria as measured by decreased length and increased width resulting in more circular or round mitochondria (*p* < 0.05, Figure 2). In axons overexpressing cytNmnat1, the total mitochondrial length and width were relatively stable over time, though they did get shorter (*p* < 0.05, Figure 2), indicating that the organelles remain filamentous overtime with cytNmnat1 overexpression. Finally, axons treated with overexpressed cytNmnat1 + vincristine were statistically similar to cells only overexpressing cytNmnat1, thus cytNmnat1 protects the cells from the vincristine induced mitochondrial fragmentation (*p* < 0.05, Figure 2). It is interesting to note that mitochondria are larger-both in length and width- in the untreated axons (*p* < 0.05, Figure 2).

### Mitochondrial Motility is Partially Maintained when Neurodegeneration is Prevented by cytNmnat1

The final aspect of mitochondrial dynamics that we studied is velocity of organelle movement by analyzing kymographs from the three treatments. Treatment with vincristine shows a trend of decreasing mitochondrial movement from 0.132 ± 0.234 μm/s at 24 h vincristine treatment to no movement at all by 96 h (Table 2). We show that mitochondria move an average of 0.191 ± 0.088 μm/s when cytNmnat1 is overexpressed for 24 h, this rate is relatively stable out to 96 h (Table 2). Overexpression of cytNmnat1 does protect from the vincristine defect bringing velocities back up to 0.083 ± 0.015 μm/s by 96 h treatment, though not up to the level of cytNmnat1 expressing axons (Figure 3, Table 2).

In summary, vincristine treatment significantly alters all measured mitochondrial parameters and cytNmnat1 expression significantly counteracts fission, fusion, and fragmentation changes in the axons of vincristine treated neurons.

**Table 2:** **Velocity of mitochondria in axons treated with vincristine and cytNmnat1 (μm/s)**.

	24 h	48 h	96 h
cytNmnat1	0.191 ± 0.088	0.168 ± 0.118	0.179 ± 0.057
Vincristine	0.132 ± 0.234	0.011 ± 0.02	0
cytNmnat1 + vincristine	0.066 ± 0.033	0.108 ± 0.039	0.083 ± 0.015

### Vincristine Alters Mitochondrial Dynamics Prior to Visible Axon Degeneration

Thus far, we have shown that cytNmnat1 overexpression, which is known to protect axons from degeneration, significantly prevents the alteration of mitochondrial dynamics as well. In an effort to understand more about the process of vincristine induced neurodegeneration, we assessed degeneration over time to determine which takes place first, the axonal morphological changes or the changes in mitochondrial dynamics. Our results indicate that degeneration of the axons induced by vincristine was detectible by 24 h but was not significantly different from cytNmnat1 or cytNmnat1 + vincristine treatments until 96 h (*F*_(2,75)_ = 11.4 *p* < 0.0001, Figure 4). As expected, throughout the time course there was no difference between the cytNmnat1 overexpressing axons and the cytNmnat1 overexpressing plus vincristine treatment.

Mitochondrial fission and fusion are significantly decreased by 24 h (Fission: *p* < 0.0116, Fusion: *p* < 0.0123, Table 1) while fragmentation in these degenerating neurons was apparent by 48 h when compared to cytNmnat1 overexpressed cells (*p* < 0.0004), (Figure 2). From these results we can conclude that vincristine treatment causes a decrease in fission and fusion, followed by mitochondrial fragmentation, then visible axon degeneration.

## Discussion

Axon degeneration, a hallmark of many neurodegenerative diseases, has been found to occur prior to clinical manifestation of symptoms and has been linked to mitochondrial dysfunction in a number of instances (Press and Milbrandt, 2008; Vohra et al., 2010). Elucidating the mechanism of axon degeneration, especially as it relates to mitochondrial dysfunction in neurodegeneration, can lead to preventative and therapeutic measures against such debilitating afflictions. Recent work has indicated that axon degeneration is an active but apoptosis-independent mechanism (Vohra et al., 2010; Gerdts et al., 2013). However, mitochondrial involvement in the form of opening of mitochondrial permeability transition pores and mitochondrial Sarm1 is implicated in axon degeneration (Barrientos et al., 2011). Furthermore, abnormal mitochondrial dynamics and altered mitochondrial motility is implicated in synaptic degeneration in the mouse models of Alzheimer’s disease (Calkins et al., 2011). In the present investigation we analyzed the relationship between mitochondria dynamics, morphology and neurodegeneration.

As expected, we show that vincristine fragments the mitochondria and decreases mitochondrial motility (Avery et al., 2012; Kitay et al., 2013). We also show that vincristine significantly disrupts mitochondrial fission and fusion. This is most likely because loss of microtubules -induced by vincristine- drastically reduces mitochondrial dynamics, most recently shown in Woods et al. (2016).

To try and understand the mechanism of neurodegeneration, we furthered our analysis by looking at the protective effect of cytNmnat1 on mitochondrial dynamics and morphology after vincristine induced neurodegeneration. Nmnat protects axons against various insults but its protective mechanism is still obscure. Nmnat protects axons in conditions of rotenone toxicity and blocks the ROS production in rotenone treated neurons (Press and Milbrandt, 2008). Nmnat overexpression also prevents the depletion of axonal NAD^+^ and maintains the levels of ATP in the transected axons (Wang et al., 2005; Press and Milbrandt, 2008; Vohra et al., 2010; Wen et al., 2011), thus suggesting that mitochondrial function may be influenced by the overexpression of Nmnat. Here we demonstrate that cytNmnat1 can prevent the loss of fission and fusion due to vincristine. We and others also show that various forms of Nmnat recover mitochondrial motility after induction of neurodegeneration, though not always significantly (Avery et al., 2012). It has also been demonstrated that this mitochondrial transport is required for Nmnat’s protective activity (Avery et al., 2012; Fang et al., 2014). Similar types of deficits in axonal mitochondrial motility have been observed in axon degeneration induced by: expression of the human mitofusion2 (Mfn2) mutant protein in the cultured DRG neurons (Baloh et al., 2007; Misko et al., 2010), hydrogen peroxide exposure and oxygen-glucose deprivation in primary hippocampal cultures (Fang et al., 2014). Cagalinec et al. (2013) also observed decreased motility, fission, and fusion in neurodegeneration disease models involving Huntingtin and Tau. Additionally, our work and that of Kitay et al. (2013) indicates that cytNmnat1 prevents mitochondrial fragmentation. The direct reason for mitochondrial fragmentation during neuron degeneration is unclear, but it is likely a result of the combined decrease in mitochondrial fission, fusion and motility.

Finally, the work presented here suggests that the decrease in fission, fusion, motility, and mitochondrial fragmentation occur prior to the axon’s morphological changes when treated with vincristine. Barrientos et al. (2011) also showed that mitochondrial swelling precedes neurodegeneration, supporting our study.

Are mitochondrial dynamics directly involved in axon degeneration? It is well established that mitochondrial function is controlled by proper morphology and distribution (Nunnari and Suomalainen, 2012) and that morphology and distribution is maintained by mitochondrial dynamics (Nunnari et al., 1997; Bleazard et al., 1999; Karbowski and Youle, 2003; Twig and Shirihai, 2011; El Zawily et al., 2014). The literature demonstrates that mitochondrial dynamics are involved in the regulation of autophagy; more specifically mutations in PINK (which cause parkinsonism and mis-regulate autophagy) induce fragmentation of the mitochondria through a block in fusion. Additionally, work on the mitochondrial motility protein -Miro- has signified that it is a substrate of PINK/parkin thus autophagy will also prevent mitochondrial motility. Thus, the loss of fission, fusion, and motility may inhibit mitochondrial function, and stimulate mitophagy.

Additionally, Wallerian degeneration is induced by a calcium overload (Wang et al., 2012) that likely activates the mitochondrial permeability transition pore (Barrientos et al., 2011). Therefore, we suggest calcium overload decreases mitochondrial dynamics (fission, fusion, and motility), inducing fragmentation and subsequently, mitochondrial function, leading to mitophagy and neurodegeneration. Avery et al. (2012) also showed something similar, demonstrating that axotomy induces a calcium overload, while Wld^S^ prevents this overload, maintains permeability resistance and mitochondrial motility. Future experiments need to focus on mitochondrial dynamics under calcium overload conditions, and neurodegeneration measurements with Mdivi-1 treatment. Mdivi-1 is an inhibitor of DRPs the regulators of mitochondrial fission and fusion. It must also be stated that the work presented here only begins to look at the role of mitochondria in neurodegeneration- specifically studying mitochondrial dynamics. At this time cytochrome c release, change in membrane potential, and the induction of apoptosis were not determined, though these are important future experiments to definitively identify the role of mitochondria in degeneration.

In conclusion, we propose that vincristine induced axon degeneration is triggered/propagated by decreased mitochondrial dynamics and fragmentation, and that cytNmnat1 inhibits axonal degeneration by preserving normal mitochondrial integrity and dynamics.

## Author Contributions

BPSV designed the project. KN, GWB, LCW acquired, analyzed, and interpreted the work. KN, GWB, LCW, BPSV, were involved in writing, editing and final approval of the version to be published. KN, GWB, LCW, BPSV agree to be accountable for all aspects of the work.

## Conflict of Interest Statement

The authors declare that the research was conducted in the absence of any commercial or financial relationships that could be construed as a potential conflict of interest.

## References

[B1] Alobuia W. M. Xia W. Vohra B. P. S. (2013). Axon degeneration is key component of neuronal death in amyloid-β toxicity. Neurochem. Int. 63, 782–789. 10.1016/j.neuint.2013.08.013 24083988 PMC3918889

[B2] Araki T. Sasaki Y. Milbrandt J. (2004). Increased nuclear NAD biosynthesis and SIRT1 activation prevent axonal degeneration. Science 305, 1010–1013. 10.1126/science.1098014 15310905

[B3] Avery M. A. Rooney T. M. Pandya J. D. Wishart T. M. Gillingwater T. H. Geddes J. W.. (2012). Wld^S^ prevents axon degeneration through increased mitochondrial flux and enhanced mitochondrial Ca^2+^ buffering. Curr. Biol. 22, 596–600. 10.1016/j.cub.2012.02.043 22425157 PMC4175988

[B4] Baloh R. H. Schmidt R. E. Pestronk A. Milbrandt J. (2007). Altered axonal mitochondrial transport in the pathogenesis of charcot-marie-tooth disease from mitofusin 2 mutations. J. Neurosci. 27, 422–430. 10.1523/JNEUROSCI.4798-06.2007 17215403 PMC6672077

[B5] Barrientos S. A. Martinez N. W. Yoo S. Jara J. S. Zamorano S. Hetz C.. (2011). Axonal degeneration is mediated by the mitochondrial permeability transition pore. J. Neurosci. 31, 966–978. 10.1523/JNEUROSCI.4065-10.2011 21248121 PMC3245862

[B6] Bleazard W. McCaffery J. King E. Bale S. Mozdy A. Tieu Q.. (1999). The dynamin-related GTPases, Dnm1, regulates mitochondrial fission in yeast. Nat. Cell Biol. 1, 298–304. 10.1038/13014 10559943 PMC3739991

[B7] Cagalinec M. Safiulina D. Liiv M. Liiv J. Choubey V. Wareski P.. (2013). Principles of the mitochondrial fusion and fission cycle in neurons. J. Cell Sci. 126, 2187–2197. 10.1242/jcs.118844 23525002

[B8] Calkins M. J. Manczak M. Mao P. Shirendeb U. Reddy P. H. (2011). Impaired mitochondrial biogenesis, defective axonal transport of mitochondria, abnormal mitochondrial dynamics and synaptic degeneration in a mouse model of Alzheimer’s disease. Hum. Mol. Genet. 20, 4515–4529. 10.1093/hmg/ddr381 21873260 PMC3209824

[B9] Canta A. Pozzi E. Carozzi V. (2015). Mitochondrial dysfunction in Chemotherapy-Induced Peripheral Neuropathy (CIPN). Toxics 3, 198–223. 10.3390/toxics3020198 PMC5634687 29056658

[B10] Chen H. Chan D. C. (2005). Emerging functions of mammalian mitochondrial fusion and fission. Hum. Mol. Genet. 14, R283–R289. 10.1093/hmg/ddi270 16244327

[B11] Chen H. Chan D. C. (2009). Mitochondrial dynamics-fusion, fission, movement and mitophagy-in neurodegenerative diseases. Hum. Mol. Genet. 18, R169–R176. 10.1093/hmg/ddp326 19808793 PMC2758711

[B12] Chen L. Xie Z. Turkson S. Zhuang X. (2015). A53T human α-synuclein overexpression in transgenic mice induces pervasive mitochondria macroautophagy defects preceding dopamine neuron degeneration. J. Neurosci. 35, 890–905. 10.1523/JNEUROSCI.0089-14.2015 25609609 PMC4300331

[B13] Dedov V. N. Roufogalis B. D. (1999). Organisation of mitochondria in living sensory neurons. FEBS Lett. 456, 171–174. 10.1016/s0014-5793(99)00951-5 10452552

[B14] El Zawily A. M. Schwarzländer M. Finkemeier I. Johnston I. G. Benamar A. Cao Y.. (2014). FRIENDLY regulates mitochondrial distribution, fusion and quality control in arabidopsis. Plant Physiol. 166, 808–828. 10.1104/pp.114.243824 25165398 PMC4213110

[B15] Fang C. Decker H. Banker G. (2014). Axonal transport plays a crucial role in mediating the axon-protective effects of NmNAT. Neurobiol. Dis. 68, 78–90. 10.1016/j.nbd.2014.04.013 24787896 PMC4106704

[B16] Gerdts J. Summers D. W. Sasaki Y. DiAntonio A. Milbrandt J. (2013). Sarm1-mediated axon degeneration requires Both SAM and TIR interactions. J. Neurosci. 33, 13569–13580. 10.1523/JNEUROSCI.1197-13.2013 23946415 PMC3742939

[B17] Karbowski M. Youle R. (2003). Dynamics of mitochondrial morphology in healthy cells and during apoptosis. Cell Death Differ. 10, 870–880. 10.1038/sj.cdd.4401260 12867994

[B18] Kitay B. M. McCormack R. Wang Y. Tsoulfas P. Zhai R. G. (2013). Mislocalization of neuronal mitochondria reveals regulation of Wallerian degeneration and NMNAT/WLDS-mediated axon protection independent of axonal mitochondria. Hum. Mol. Genet. 22, 1601–1614. 10.1093/hmg/ddt009 23314018 PMC3657477

[B19] Korobova F. Ramabhadran V. Higgs H. N. (2013). An actin-dependent step in mitochondrial fission mediated by the ER-associated formin INF2. Science 339, 464–467. 10.1126/science.1228360 23349293 PMC3843506

[B20] Liesa M. Palacín M. Zorzano A. (2009). Mitochondrial dynamics in mammalian health and disease. Physiol. Rev. 89, 799–845. 10.1152/physrev.00030.2008 19584314

[B21] Martin L. J. (2012). “Chapter 11–biology of mitochondria in neurodegenerative diseases,” in Progress in Molecular Biology and Translational Science, ed. David B. T. (Cambridge, UK: Academic Press), 355–415. 10.1016/B978-0-12-385883-2.00005-9 PMC3530202 22482456

[B22] Misko A. Jiang S. Wegorzewska I. Milbrandt J. Baloh R. H. (2010). Mitofusin 2 is necessary for transport of axonal mitochondria and interacts with the miro/milton complex. J. Neurosci. 30, 4232–4240. 10.1523/JNEUROSCI.6248-09.2010 20335458 PMC2852190

[B23] Müller M. Mironov S. L. Ivannikov M. V. Schmidt J. Richter D. W. (2005). Mitochondrial organization and motility probed by two-photon microscopy in cultured mouse brainstem neurons. Exp. Cell Res. 303, 114–127. 10.1016/j.yexcr.2004.09.025 15572032

[B25] Nunnari J. Marshall W. Straight A. Murray A. Sedat J. Walter P. (1997). Mitochondrial transmission during mating in S. cerevisiae is determined by mitochondrial fusion and fission and the intramitochondrial segregation of mtDNA. Mol. Biol. Cell 8, 1233–1242. 10.1091/mbc.8.7.1233 9243504 PMC276149

[B24] Nunnari J. Suomalainen A. (2012). Mitochondria: in sickness and in health. Cell 148, 1145–1159. 10.1016/j.cell.2012.02.035 22424226 PMC5381524

[B26] Press C. Milbrandt J. (2008). Nmnat delays axonal degeneration caused by mitochondrial and oxidative stress. J. Neurosci. 28, 4861–4871. 10.1523/JNEUROSCI.0525-08.2008 18463239 PMC2678678

[B27] Rambold A. S. Kostelecky B. Elia N. Lippincott-Schwartz J. (2011). Tubular network formation protects mitochondria from autophagosomal degradation during nutrient starvation. Proc. Natl. Acad. Sci. U S A 108, 10190–10195. 10.1073/pnas.1107402108 21646527 PMC3121813

[B28] Sasaki Y. Vohra B. P. S. Lund F. E. Milbrandt J. (2009). Nicotinamide mononucleotide adenylyl transferase-mediated axonal protection requires enzymatic activity but not increased levels of neuronal nicotinamide adenine dinucleotide. J. Neurosci. 29, 5525–5535. 10.1523/JNEUROSCI.5469-08.2009 19403820 PMC3162248

[B29] Schimmel B. Berbusse G. Naylor K. (2012). Mitochondrial fission and fusion in Dictyostelium discoideum: a search for proteins involved in membrane dynamics. BMC Res. Notes 5:505. 10.1186/1756-0500-5-505 22980139 PMC3492061

[B30] Shen Q. Yamano K. Head B. P. Kawajiri S. Cheung J. T. M. Wang C.. (2014). Mutations in Fis1 disrupt orderly disposal of defective mitochondria. Mol. Biol. Cell 25, 145–159. 10.1091/mbc.E13-09-0525 24196833 PMC3873885

[B31] Summers D. W. DiAntonio A. Milbrant J. (2014). Mitochondrial dysfunction induces Sarm-1dependent cell death in sensory neurons. J. Neurosci. 34, 9338–9350. 10.1523/JNEUROSCI.0877-14.2014 25009267 PMC4087211

[B33] Twig G. Elorza A. Molina A. J. A. Mohamed H. Wikstrom J. D. Walzer G.. (2008). Fission and selective fusion govern mitochondrial segregation and elimination by autophagy. EMBO J. 27, 433–446. 10.1038/sj.emboj.7601963 18200046 PMC2234339

[B32] Twig G. Shirihai O. S. (2011). The interplay between mitochondrial dynamics and mitophagy. Antioxid. Redox Signal. 14, 1939–1951. 10.1089/ars.2010.3779 21128700 PMC3078508

[B34] Vohra B. P. S. Sasaki Y. Miller B. R. Chang J. DiAntonio A. Milbrandt J. (2010). Amyloid precursor protein cleavage-dependent and -independent axonal degeneration programs share a common nicotinamide mononucleotide adenylyltransferase 1-sensitive pathway. J. Neurosci. 30, 13729–13738. 10.1523/JNEUROSCI.2939-10.2010 20943913 PMC3104322

[B36] Wang J. T. Medress Z. A. Barres B. A. (2012). Axon degeneration: molecular mechanisms of a self-destruction pathway. J. Cell Biol. 196, 7–18. 10.1083/jcb.201108111 22232700 PMC3255986

[B35] Wang J. Zhai Q. Chen Y. Lin E. Gu W. McBurney M. W.. (2005). A local mechanism mediates NAD-dependent protection of axon degeneration. J. Cell Biol. 170, 349–355. 10.1083/jcb.200504028 16043516 PMC2171458

[B37] Wen Y. Parrish J. Z. He R. Zhai R. G. Kim M. D. (2011). Nmnat exerts neuroprotective effects in dendrites and axons. Mol. Cell. Neurosci. 48, 1–8. 10.1016/j.mcn.2011.05.002 21596138 PMC3152617

[B38] Woods L. C. Berbusse G. W. Naylor K. (2016). Microtubules are essential for mitochondrial dynamics-fission, fusion and motility-in dictyostelium discoideum. Front. Cell Dev. Biol. 4:19. 10.3389/fcell.2016.00019 27047941 PMC4801864

